# Integrating technologies provides insight into the subsurface foraging behaviour of a humpback whale (*Megaptera novaeangliae*) feeding on walleye pollock (*Gadus chalcogrammus*) in Juan de Fuca Strait, Canada

**DOI:** 10.1371/journal.pone.0282651

**Published:** 2023-03-06

**Authors:** Rhonda Reidy, Stéphane Gauthier, Thomas Doniol-Valcroze, Matthew A. Lemay, Rute B. G. Clemente-Carvalho, Laura L. E. Cowen, Francis Juanes

**Affiliations:** 1 Department of Biology, University of Victoria, Victoria, British Columbia, Canada; 2 Institute of Ocean Sciences, Fisheries and Oceans Canada, Sidney, British Columbia, Canada; 3 Pacific Biological Station, Fisheries and Oceans Canada, Nanaimo, British Columbia, Canada; 4 Hakai Institute Genomics Laboratory, Quadra Island, British Columbia, Canada; 5 Department of Mathematics and Statistics, University of Victoria, Victoria, British Columbia, Canada; MARE – Marine and Environmental Sciences Centre, PORTUGAL

## Abstract

Subsurface foraging is an important proportion of the activity budget of rorqual whales, yet information on their behaviour underwater remains challenging to obtain. Rorquals are assumed to feed throughout the water column and to select prey as a function of depth, availability and density, but there remain limitations in the precise identification of targeted prey. Current data on rorqual foraging in western Canadian waters have thus been limited to observations of prey species amenable to surface feeding, such as euphausiids and Pacific herring (*Clupea pallasii*), with no information on deeper alternative prey sources. We measured the foraging behaviour of a humpback whale (*Megaptera novaeangliae*) in Juan de Fuca Strait, British Columbia, using three complimentary methods: whale-borne tag data, acoustic prey mapping, and fecal sub-sampling. Acoustically detected prey layers were near the seafloor and consistent with dense schools of walleye pollock (*Gadus chalcogrammus*) distributed above more diffuse aggregations of pollock. Analysis of a fecal sample from the tagged whale confirmed that it had been feeding on pollock. Integrating the dive profile with the prey data revealed that the whale’s foraging effort followed the general pattern of areal prey density, wherein the whale had a higher lunge-feeding rate at the highest prey abundance and stopped feeding when prey became limited. Our findings of a humpback whale feeding on seasonally energy-dense fish like walleye pollock, which are potentially abundant in British Columbia, suggests that pollock may be an important prey source for this rapidly growing whale population. This result is informative when assessing regional fishing activities for semi-pelagic species as well as the whales’ vulnerability to fishing gear entanglements and feeding disturbances during a narrow window of prey acquisition.

## Introduction

Foraging opportunities in the ocean are distributed across space and time in ‘biotic patchiness’ [1]. For a migrating baleen whale (Mysticeti), food may predictably be available on a feeding ground that spans hundreds or even thousands of square kilometers (e.g., North Atlantic right whales [2], North Pacific blue whales [3]). Within the feeding ground, however, aggregations of zooplankton and small fish, such as krill (e.g., *Euphausia* spp.), herring (*Clupea pallasii*) and anchovies (*Engraulis mordax*), which serve as food ‘patches’ for baleen whales, are relatively unpredictable on a day-to-day basis in terms of location, timing and density. The spatial and temporal patterns of food patches on feeding grounds are regulated by the biotic (e.g., primary production) and abiotic (e.g., turbulent mixing) processes at any given area [4], and range over scales of a few square kilometers to only a few square meters within the larger niche space [4,5]. A highly mobile rorqual whale (*Balaenopteridae*), such as a humpback whale (*Megaptera novaeangliae*), thus likely draws on a broad spectrum of sensory information, including visual, acoustical, and tactile signals [6,7], as well as memory of profitable feeding sites [8] to inform its foraging decisions at multiple levels [9]. Sensory information is likely vital to a rorqual finding patchy food in a vast ocean seascape, but precisely how the whale responds to cues about prey in its environment [6], or what it may choose to eat at any given time, remains largely elusive.

Previous analyses from whale-borne tag and video data indicate that rorqual species employ an efficient underwater engulfment-filtration strategy, and may consume an entire prey school in a single gulp [10–13]. While spatial associations of the feeding rorquals and their prey distribution and abundance are often quantified using fisheries active acoustics techniques, primarily with scientific echosounders [7,10,14–17], there remain limitations in the precise identification of the organisms in the water column without additional net sampling [18,19], which often is not included for logistical reasons and the high operating costs of trawling [20]. Nevertheless, the evidence for rorquals’ significant role in ocean food webs is substantial [21,22], and field studies continue to alter our perception of rorquals as adaptable predators to a dynamic range of prey depth [23], availability [15,24], densities [16,25–28] and ocean climates [29].

The underwater foraging strategy of rorquals has required consideration of complicated measures such as the biomechanics during bulk prey collection [13,30]. As gigantic filter feeders, the whales’ vast engulfment capacities [11] and raptorial-like lunge feeding behaviour [10,30] have evolved to overwhelm the escape responses of swarming zooplankton and schooling fish [31] and maximize the amount of prey obtained in a single lunge [12]. This dramatic engulfment of prey-laden water is defined by a peak in acceleration just before the whale’s mouth opens widely, followed by rapid deceleration due to drag from the whale coasting with mouth agape and causing a distension of its buccal cavity [13,30]. The acceleration and engulfment phases of a lunge-feeding rorqual are strongly signalled in whale-borne movement sensor data, and prey-dependent kinematic signatures may occur that distinguish krill or fish feeding behaviour [32].

In the eastern North Pacific, increasing numbers of humpback whales are using Juan de Fuca Strait for feeding, with many expanding their range into historical feeding areas in the Salish Sea [33]. Located between U.S. Washington State and Vancouver Island, British Columbia, Canada, Juan de Fuca Strait is a submarine valley [34] with an unobstructed connection to the Pacific Ocean and to a highly productive continental shelf system [1,34]. Humpback whales are known to aggregate near the mouth of Juan de Fuca Strait [35,36], likely because of the area’s high biological productivity [37]. A steady increase of humpback whales in the Strait has been documented in recent years as the population has grown [38]. In British Columbia, contemporary data on humpback whale feeding behaviour have been limited to surface observations, largely due to the financial and logistical challenges of studying whales. Though informative, the data obtained by surface observation may be biased toward prey types amenable to surface feeding (e.g., euphausiids (krill), Pacific herring), with no information on the whales’ feeding performance on deeper aggregations and alternative prey sources [14,15]. Deeper, subsurface foraging is an important proportion of the activity budget of all rorquals [39], and when the whales are in western Canadian waters, substantial knowledge gaps remain concerning underwater feeding behaviour and prey choices [40,41].

A detailed description of the diving behaviour of humpback whales as it relates to prey in Juan de Fuca Strait is needed to better understand the feeding strategies of these whales, especially given that they are increasingly foraging in a region of heavy marine traffic and ship strike risk for large whales [36,42]. This information can be used to determine minimum prey density requirements and habitat suitability [43] as well as modelling the potential impacts of anthropogenic stressors [44]. Here we present the first account of deep foraging behaviour of a humpback whale in the Canadian waters of Juan de Fuca Strait, by simultaneously integrating three intensive sampling tools: whale-borne tag data, acoustic prey mapping, and microscopy and DNA metabarcoding of a fecal sample from the tagged whale. We show that the foraging effort of the whale followed the general pattern of patchy prey located near the seafloor, and that the fecal sample from the whale provided information that was consistent with the acoustic prey results.

## Materials and methods

### Study location

**Fig 1:**
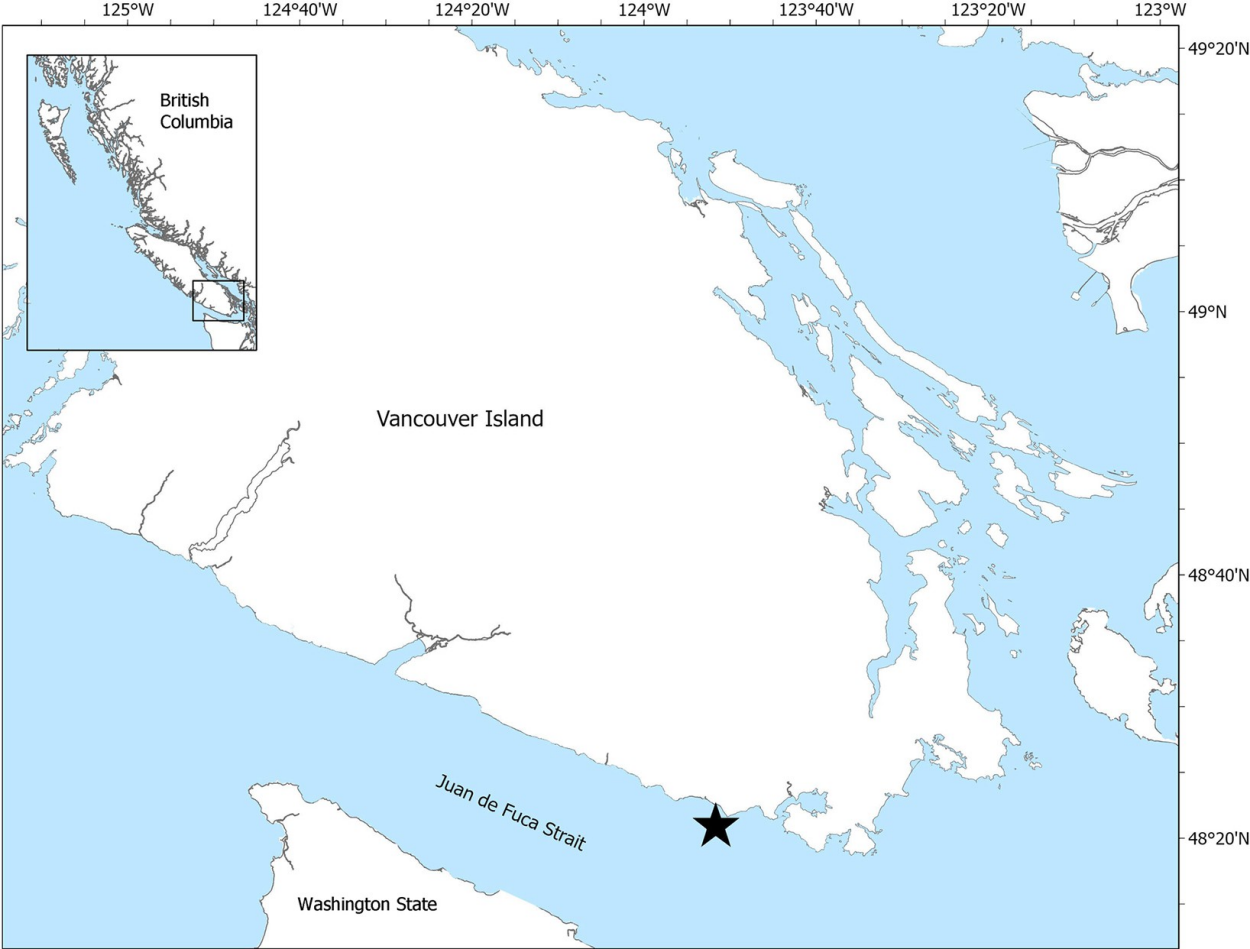
Map of southern Vancouver Island, British Columbia, Canada showing the study site (black star) in Juan de Fuca Strait. Basemap was sourced from the BC Data Catalogue and contains information licensed under the Open Government Licence–British Columbia (https://catalogue.data.gov.bc.ca/dataset/freshwater-atlas-islands).

All data for the three collection schemes (tagging, acoustic prey mapping and fecal sampling) were collected from an 8-m aluminum boat in the Canadian waters of Juan de Fuca Strait in September 2017, approximately one mile offshore of Otter Point, Vancouver Island, British Columbia (Fig 1). Juan de Fuca Strait is a narrow submarine valley roughly 160 by 20 km, located between southern Vancouver Island and the Olympic Mountains of Washington State [34]. The western end of the Strait provides a relatively unobstructed connection to deeper waters of the Pacific Ocean, where denser, nutrient-rich water flows into the Strait and is tidally mixed with outflowing fresher surface water [34]. Data were collected in an area that humpback whales are regularly observed subsurface feeding in singles and pairs. All work was conducted under Marine Mammal License MML-45, and approved by the University of Victoria’s Animal Care Committee under protocol 2017–009.

### Data collection

We used a multi-sensor suction-cup tag (Customized Animal Tracking Solutions, CATS, www.cats.is) to collect high sample rate kinematic and behavioural data from a foraging humpback whale. The whale was actively engaged in feeding underwater and was easily approached while recovering from a dive. The tag was attached near the dorsal fin using a slow vessel approach from behind and to the side of the whale, and a 7-m handheld carbon fiber pole. The whale returned to its pre-approach feeding behaviour within seconds of tag attachment. The tag contained a 3-axis magnetometer, gyroscope, and accelerometer sampling at 20 Hz, and a pressure sensor sampling at 10 Hz. The tag also contained a VHF transmitter that enabled close tracking of the whale while the tag was attached. After a pre-set release time of 4 hours, the tag detached from the whale and was recovered for downloading the data.

The vertical distribution of mesozooplankton and fish was continuously recorded near the tagged whale using an Acoustic Zooplankton and Fish Profiler (AZFP) from ASL Environmental Sciences, Victoria, British Columbia. The AZFP is an autonomous scientific echosounder, designed for long-term monitoring of the water column from a mooring on the seafloor. We tested the portability of the AZFP in a vessel-mounted, downward-looking orientation from the sea surface. The transducers were mounted on a metal strut and lowered over the side of the boat to 1-m water depth, while the instrument in its pressure case remained on the boat. We used individually calibrated 125 and 200 kHz channels (7° and 10° conical beams) that transmitted sequentially, providing an acoustic sample every two seconds at a pulse duration of 300 μs (Table 1). Power levels of the AZFP were well below the levels emitted by a hull-mounted system typically used in mobile acoustic surveys (e.g., [45]), while the 125 and 200 kHz frequencies are well above the estimated hearing range of humpback whales (0.02–24 kHz [46]). Volume backscatter data (S_v,_ dB) were recorded and stored by the instrument in Compact FLASH memory. Acoustic data were corroborated using regional information from Fisheries and Oceans Canada multi-year, integrated trawl and acoustic survey data on Pacific hake (*Merluccius productus*) and Strait of Georgia pelagic ecosystem surveys [47,48].

**Table 1:** Acoustical parameter settings for the AZFP. Units of source level (SL) are dB re 1 uPa @ 1 m.

Frequency	θ (°)	Power (W)*	SL (dB)*	Pulse duration (µs)	Ping rate (s)
125	7	14	211.0	300	2
200	10	26	210.6	300	2

*Values from S. Pearce, ASL Environmental Sciences, pers. comm.

Upon tag attachment to the whale, acoustic prey sampling was initiated to record whole water column data from the surface to the seafloor within 10 to 200 m of the tagged whale during the period of tag data logging. The whale was followed at 1.0–2.6 m s^-1^ (2–5 knots) based on surface observations with the acoustic survey track assumed to follow the general swimming track of the whale. Continuous GPS positions were recorded at 0.5 s intervals by a handheld Garmin GPS, while periodic GPS surfacing locations were noted, based on either the boat’s position when close to the surfacing whale, or on the whale’s fluke print location (a calm patch of water created by the diving whale). The AZFP and handheld GPS clocks were matched at the start and end of the deployment, while surface observations were manually logged and time-synchronized with the GPS clock, and continued until the tag was released from the whale.

### Tag data analysis

Tag orientation on the whale was corrected and animal orientation in the water was calculated using custom-written scripts in Matlab (2014a; Natick, Massachusetts: MathWorks, Inc.) (following [32,49]). The whale’s reference frame was thus *x* (longitudinal), *y* (lateral) and *z* (dorso-ventral). The whale’s dive profile was categorized into three phases: descent, bottom foraging, and ascent, to distinguish the decent/ascent phases of diving from feeding [50] prior to comparing with prey distribution data. We calculated the descent and ascent times for the entire data set using pitch and water depth parameters. Descent time was defined by cut-off thresholds between 0 m at the surface and the depth at which the first zero pitch angle occurred, signalling that the animal was no longer descending. Ascent started at the first pitch angle ≥ +40° with subsequent decreasing depth and ended when depth equaled zero. Foraging time was defined as the time between the end of a descent and the start of an ascent, and recovery time at the surface was the time between the end of an ascent and the start of the next dive [50]. Animal speed was estimated from changes in depth over time and turbulent flow that vibrated the tag (tag “jiggle” [51]). Lunge feeding events were identified from stereotypical kinematic spikes in speed, pitch angle and tag jiggle amplitude (tag “jerk”) using custom lunge-audit scripts in Matlab 2017b [32,51]. A lunging rorqual typically accelerates toward prey using strong tail thrusts (fluking) and coincident quick changes in body orientation (e.g., pitch, roll and heading), immediately followed by rapid deceleration at the time of mouth opening [13,32]. The duration between these signal maxima represented the time interval between consecutive lunges. We averaged the signal maxima across lunges to obtain an overall estimate of mean lunge speed and pitch for the deployment. Fluking behaviour before each lunge was visually inspected using a custom-written application [52] in R software (v. 4.1.1; R Core Team, 2021) that highlighted peaks in oscillatory frequency (fluke strokes as *f*, 1/period) along the y-axis of the gyroscope signal [53]. To visualize a pseudotrack of the whale’s underwater behaviour, the combined animal orientation and GPS data were imported into Trackplot software (v. 2.3) [54].

### Acoustic data analysis

AZFP data were processed using Echoview (v.12; Echoview Software Pty Ltd.). Mean volume backscattering strength (S_v_ in dB re 1 m^-1^), a relative measure of density, was analyzed from 10 m below the surface to 5 m above the sounder-detected seafloor in bins of 5 m vertically by 5 pings horizontally. Sound speed [55] and absorption coefficients [56] were estimated at each frequency using temperature and salinity values reported for the closest oceanographic sampling station in Juan de Fuca Strait in September 2017 (approximately mid-Strait off Sooke Basin), as measured by Fisheries and Ocean Canada. Background noise was removed following the approach described in de Robertis & Higginbottom [57], using a minimum signal-to-noise ratio of 10 dB and maximum noise threshold of -125 dB re 1 m^-1^. Removal of acoustic noise was done through visual inspection of the echograms and applying filters following Ryan et al. [58]. Impulse and transient noise were removed with a maximum threshold of 10 dB and 12 dB, respectively. Backscatter in the processed echograms was scrutinized and classified based on echo morphology (shape and structure of the aggregations), depth distribution (including bottom association), and single target attributes. Mean volume backscattering strength (S_v_) at 125 kHz was subtracted from that at 200 kHz to assess differences (MVBS_200-125_). Values greater or equal to 0 dB or lower or equal to 4 dB would be indicative of swimbladder-bearing fish, while MVBS _200–125_ values greater than 5 dB would indicate backscatter dominated by zooplankton [59]. The processed volume scattering data were gridded into 1-min horizontal cells by 10-m vertical cells, then echo-integrated using an integration threshold of -70 dB over the whole water column into nautical area scattering coefficients (NASC; m^2^ nmi^-2^; [60]) to obtain relative measures of water column biomass where the whale was feeding. The overall pattern of log_10_-transformed NASC and the whale’s feeding rate during tag attachment were plotted in R, using a generalized additive model (GAM) with integrated smoothness estimation (y~s(x)). To estimate biomass per lunge count, we averaged NASC for each bottom-foraging time interval, and further averaged NASC across foraging intervals that had matching lunge counts to obtain a single estimate in each lunge-count category.

### Fecal analysis

A fecal sample was collected from the tagged whale within 1 m of the sea surface using a pool skimmer net with 0.15 mm mesh size. The sampled material was a dilute, rapidly sinking purple liquid that comprised numerous tiny fish scales and bones. The sample was placed into a new Ziploc bag, double bagged, and stored in a cooler on the boat containing a small ice block and then transferred to a -20°C freezer on shore. A 10-ml representative subsample of the feces was preserved in 95% ethanol in a sterile screw cap tube and also stored at -20°C.

The frozen fecal sample was thawed and rinsed through a 0.5 mm sieve, with the remaining hard parts cleaned in a solution of detergent and distilled water for visual identification to the lowest possible taxon using a reference collection of prey species skeletons. For the ethanol preserved sample, a DNA metabarcoding approach (amplicon sequencing) was used to infer the identity of prey items. We targeted a short fragment of the cytochrome C oxidase subunit I gene (COI) to detect marine invertebrates [61] and used the MiFish region [62] of the 12S rRNA gene to detect fish in the bulk fecal sample. All DNA extraction, genetic library preparation, sequencing, and bioinformatics were conducted at the Hakai Institute Genomics Laboratory, as one element in a larger, collaborative humpback fecal study for whales feeding in southern British Columbia [63] (see S1 Text for full DNA analysis methods).

## Results

### Tagged whale

**Fig 2:**
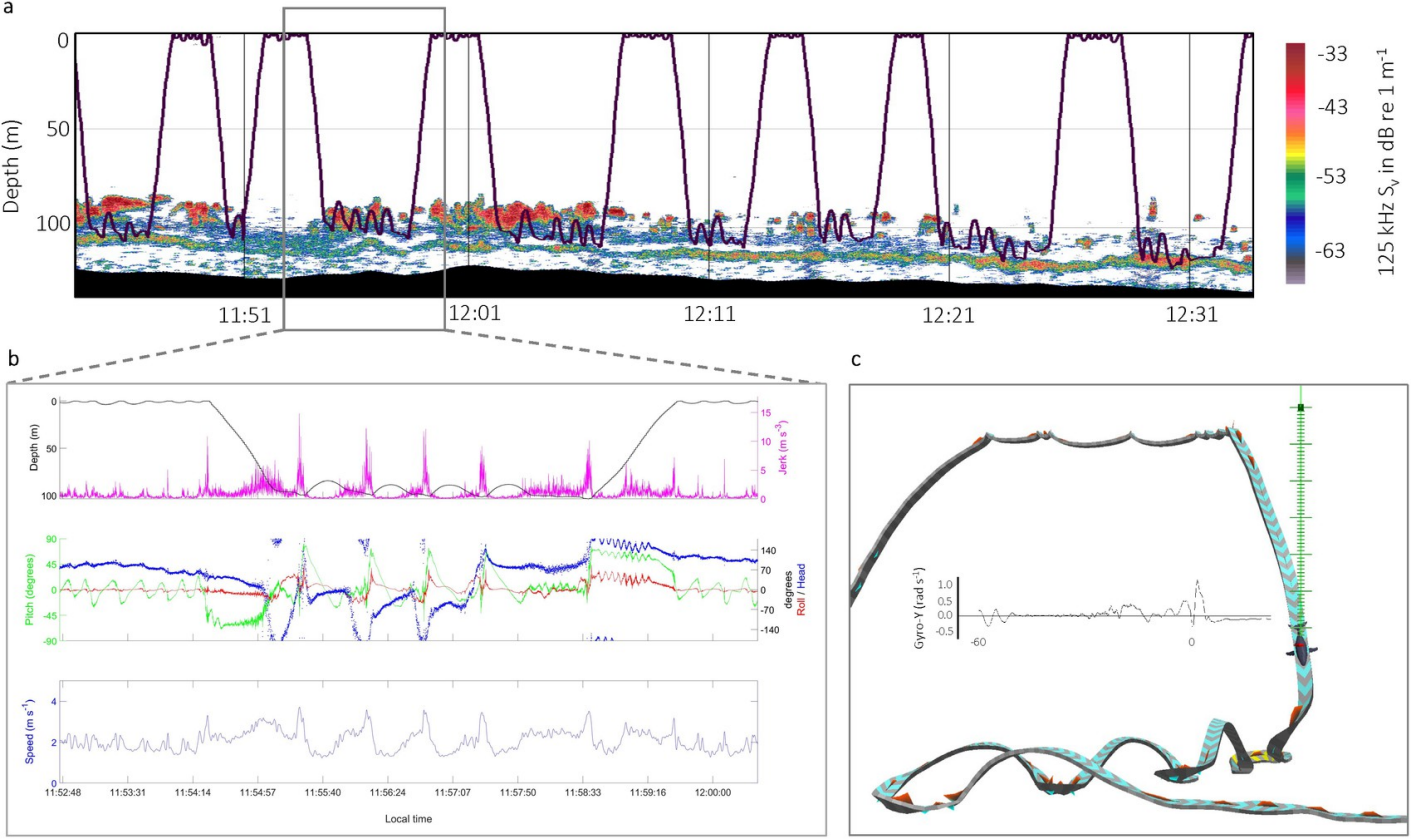
Examples of field measurements informing our estimation of underwater whale foraging behaviour and prey characteristics. (a) The dive profile of BCY0983 (black line) overlaid on a 125 kHz echogram showing prey vertical distribution and aggregation structure. The x-axis is time of day, and the left y-axis is the water depth in meters. The seafloor is black, and acoustic backscatter from prey is colour coded from gray (low echo return) to red (strong echo return) on the right legend. The black line illustrates the whale’s steep foraging dives, from the sea surface to ~100 m depth. (b) Higher kinematic signals during lunge feeding. Top plot: Rate of acceleration (jerk; pink); Middle: Pitch (green), roll (red), heading (blue); Bottom: Speed. (c) The Trackplot pseudotrack of BCY0983 corresponding to the kinematic data for the foraging dive in (b). The green vertical dashed line represents water depth, where the black dot represents the sea surface and the other end of the line shows the location of the whale on the dive track. Each horizontal line indicates 1 m, and the larger cross bars represent 10 m. The red triangles and yellow colour on the track represent fluke strokes and banked turns, respectively. The black line at the zero line of the inset graph (y-axis gyroscope in rad/s) corresponds to a 1-min example of fluking leading into the first lunge.

The tagged humpback whale was an animal previously photographed in British Columbia waters and catalogued as BCY0983 (T. Shaw, Humpback Whales of the Salish Sea sightings database, Duncan, British Columbia, unpublished data). The age and sex of the whale is unknown; however, the whale has documented fidelity to southern Vancouver Island since 2016, and is known locally as ‘Aerie’. Aerie was in Juan de Fuca Strait in our study region on days immediately before and after being tagged. All feeding activity occurred underwater in a spatially restricted surface area of < 2 km^2^, with 21 foraging dives and 81 lunges at depth recorded over 2.8 h of tag attachment data (Table 2; Fig 2A).

**Table 2:** Estimated foraging parameters for the tagged humpback whale BCY0983. Values are mean ± standard deviation and maximum (in parentheses).

Number of foraging dives	21	Number of lunges detected	81	Total time (h)	2.8
Descent duration (min)	0.8 ± 0.1 (1.0)	Lunges per dive	4 ± 1 (6)	Descent phase (min)	16.7
Descent end depth (m)	98 ± 6 (114)	Lunge depth (m)	110 ± 8 (134)	Ascent phase (min)	21.6
Descent speed (m s-1)	2.7 ± 0.2 (4.3)	Lunge interval (min)	1.0 ± 0.6 (4.3)	At surface (min)	42.3
Descent angle (deg)	-48 ± 4 (-78)	Lunge speed (m s-1)	3.7 ± 0.3 (4.7)	At foraging depth (h)	1.5
Ascent duration (min)	1.0 ± 0.2 (1.4)	Lunge angle (deg)	71 ± 13 (89)	At foraging depth/dive (min)	4.58 ± 1.78 (7.72)
Ascent speed (m s-1)	2.3 ± 0.2 (4.0)			At surface/dive interval (min)	2.06 ± 0.53 (3.52)
Ascent angle (deg)	54 ± 6 (89)				

The whale consistently dived to a mean foraging depth of 98±6 m, with a mean speed, descent angle (pitch) and duration of the descent of 2.7±0.2 m s^-1^, -48±4° and 0.8±0.1 min, respectively (Table 2). Time spent at the foraging depth on a dive (4.78±1.72 min) increased toward the end of the tag deployment (max 7.72 min). Time recovering at the surface between dives (2.06±0.53 min) also increased toward the end of the deployment, with a maximum recovery time (3.52 min) corresponding to the maximum time at depth. Nearly all ascents back to the surface from the foraging depth had only slightly lower speed, longer duration and steeper pitch than descending dives. However, ascent pitch became shallower toward the end of the deployment, with the final ascent having the lowest mean pitch at 35°.

The number of lunge-feeding events detected at the foraging depth ranged from 0 to 6 lunges per dive (mean 4±1) with a mean interval of 1.0±0.6 min between consecutive lunges. A higher feeding rate occurred within the first two hours of the deployment (37 lunges/h) that gradually decreased until the end of the data detection with no lunges detected in the last dive period. The final lunge on many dives occurred on the ascent to the surface (Fig 2B), implying active water filtration through the early part of ascent, as previously documented in other studies [23,64,65]. Mean lunge speed 3.7±0.3 m s^-1^ and pitch 71±13° were greater than in the descent/ascent phases of diving, with the highest pitch segment on a lunge prone to gimbal lock due to Euler rotation, where roll (*x*) and heading (*z*) were driven into parallel configuration with pitch (*y*). Maximum lunge speed was always attained on lunge approach. Maximum pitch occurred slightly after maximum speed and coincided with a large deceleration at the assumed moment of mouth opening (Fig 2B). At the higher feeding rate, the sensors suggest 1–3 strong fluke strokes leading to a lunge, which may have terminated in a dramatic head thrust simultaneous with the lowering of the mandibles (W. Gough, pers. comm.; [30]), and a concurrent slight negative pitch and quick role and heading changes, all suggesting a downward, circling maneuver just before a rostrum-up attack on prey (Fig 2B and 2C).

Overall, half of the tag deployment time (1.5 h) was spent at the foraging depth. The remaining time was spent recovering at the sea surface (42 min) and in the descent and ascent phases of diving (38 min).

### Acoustic prey detection

Prey were observed in echograms as two discreet layers distributed near the bottom (<150 m; Fig 2A). The top layer had a clumpy and heterogenous appearance with defined edges, consistent with fish schools that extended over a larger distance than the secondary layer, which extended in a narrow, distinctively bounded and discontinuous band (<10 m in thickness) located between the schools and the seafloor. Both layers exceeded -70 dB re 1 m^-1^ at the two frequencies, although the 200 kHz data indicated a small number of other scatterers consistent with diffuse zooplankton (weaker S_v_) that extended down to the bottom echo, making discrimination of backscatter below the schools more challenging. Numerous small individual targets were detectable on the edge and the outside of both layers. The mean dB difference for the top layer (2.96±2.58 dB) indicated a consistency with small schools of fish above a secondary, more diffuse layer of fish (1.41±2.13 dB). The vertical distribution and acoustic properties of the layers were compared with the backscatter recorded in regional acoustic-trawl surveys conducted off the west coast of Vancouver Island and in the Strait of Georgia [47,48]. In those surveys generally, two similarly distinct, near-seafloor layers of survey backscatter at 38–200 kHz frequencies were attributed almost entirely to walleye pollock (*Gadus chalcogrammus*). The trawl catches in those surveys comprised a mix of adult and juvenile walleye pollock, depending on survey year, and points to walleye pollock in the current study.

**Fig 3:**
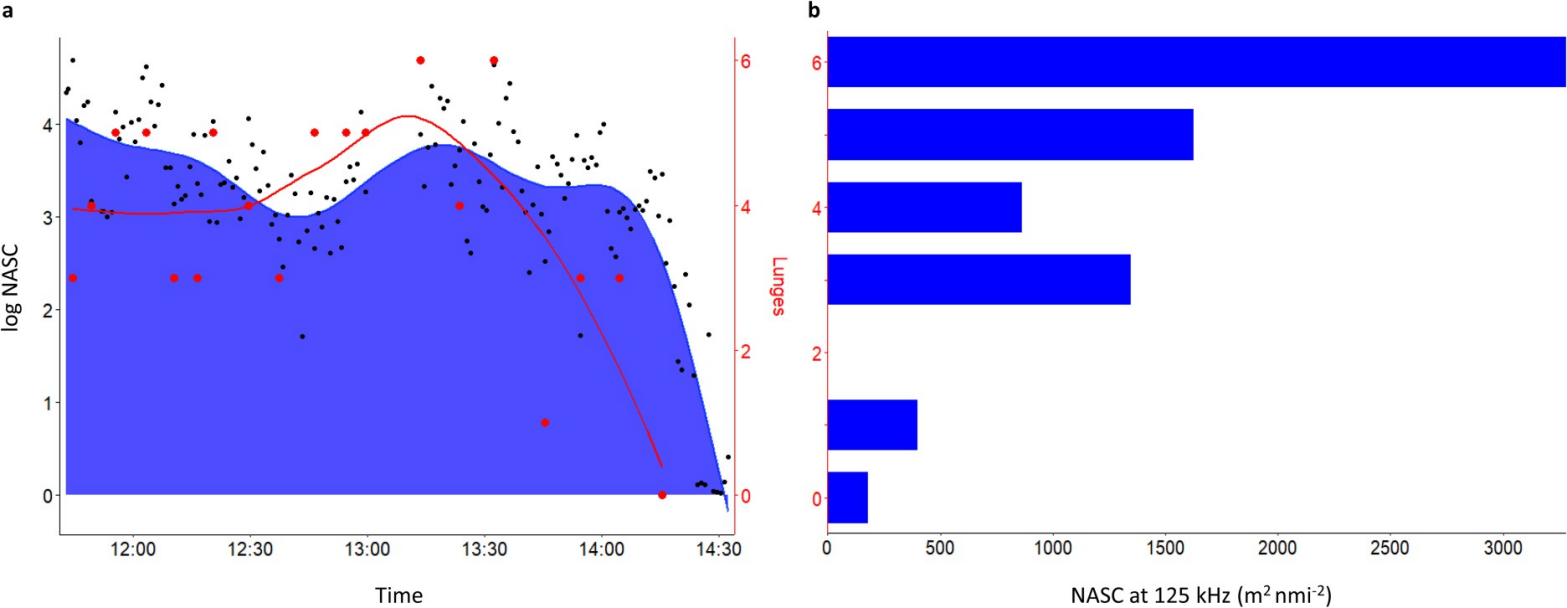
Visualization of relative water column biomass and whale foraging effort. (a) The number of whale feeding lunges per dive (red; right y-axis) overlaid on log-transformed NASC (blue; left y-axis) and smoothed, showing that the foraging effort of BCY0983 followed the general pattern of NASC over the course of the deployment. Black dots indicate: Individual NASC estimates at 1-min intervals; red dots: The number of lunges per foraging dive. (b) Histogram of the untransformed NASC estimates averaged per lunge count category; none of the foraging dives comprised 2 lunges.

Nautical Area Scattering Coefficients (NASC) in the current study were also extremely patchy, with an overall mean and maximum value at 125 kHz of 433 and 42,922 m^2^ nmi^-2^, respectively. NASC was highest in the first two hours, then declined toward zero by the end of the data detection. Overlaying the whale dive profile onto the echograms indicated that the whale lunged from below a prey aggregation and likely targeted the densest patches (Fig 2A). The whale’s foraging effort also followed the general pattern of NASC, where the number of feeding lunges per dive declined to zero when NASC declined to below ~500 m^2^ nmi^-2^ (Fig 3). Longer foraging dives also coincided with the decline in areal density (NASC; m^2^ nmi^-2^). The longest dive occurred immediately before the final dive in the deployment, which had the lowest prey density and no feeding lunges detected.

### Fecal analysis

**Fig 4:**
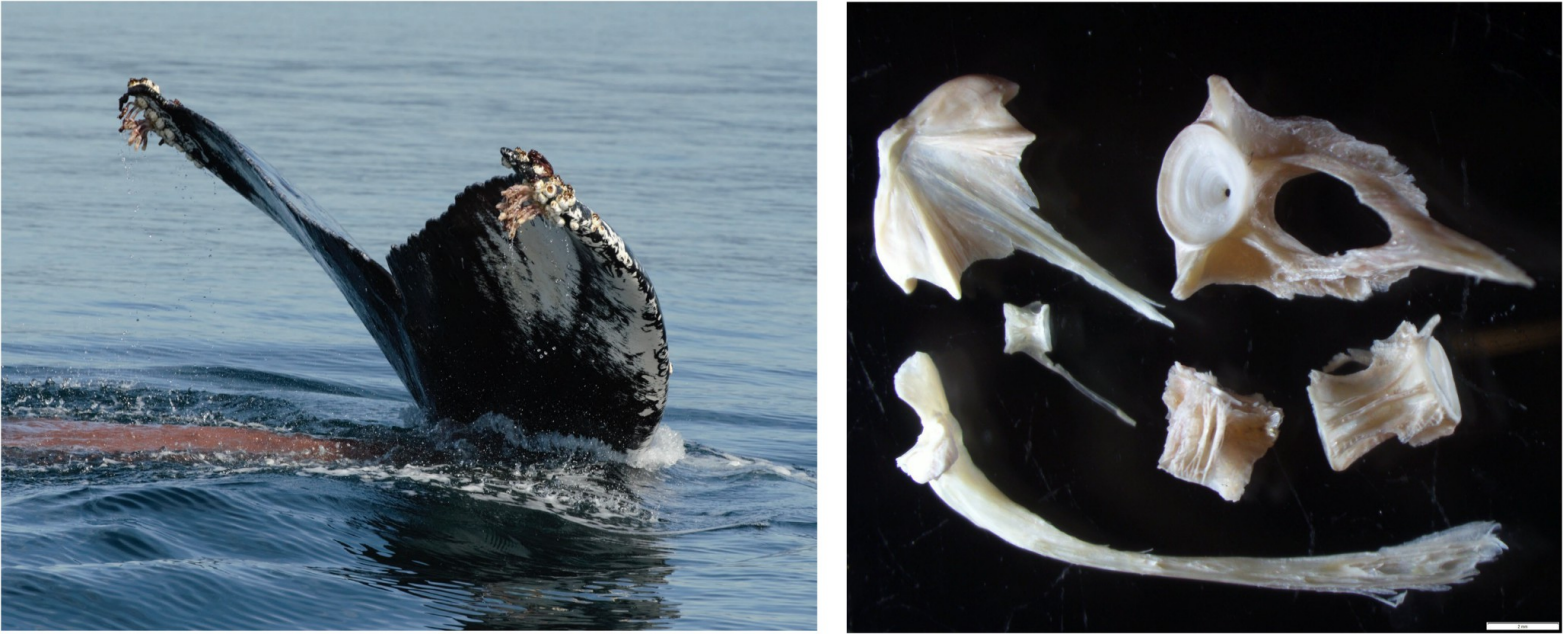
Juvenile pollock bones in the fecal sample obtained from tagged humpback whale BCY0983. Left photo: Purplish fecal plume on the water surface as the whale dived. Right: Vomer, maxilla and vertebrae of walleye pollock. Scale bar = 2 mm.

Microscope examination of fecal hard parts revealed numerous vertebrae and cranial bones from walleye pollock (Fig 4). No otoliths were present in the sample for age determination; however, vertebral sizes suggest juvenile pollock.

The genetic analysis of fish DNA in the fecal sample produced 295,543 quality-filtered 12S reads for diet analysis. A total of 180,624 (61%) of these reads were attributed to the humpback whale. From the remaining sequence data, the overwhelming majority (114,880 reads; 99.9% of fish sequences) belonged to amplicon sequence variants (ASVs) that annotated to the genus *Gadus* (see S1 Table for taxonomic assignment). These ASVs were 100% similar to public reference sequences from *Gadus chalcogrammus* (walleye pollock). Notably, there is very little genetic differentiation at this marker among closely related *Gadus* species, and these data also had a high degree of sequence similarly with *Gadus macrocephalus* (99%), which makes it challenging to provide a definitive species-level assignment based on genetics alone. However, in combination with the acoustic data and microscopy, these genetic data support the hypothesis that the whale was targeting aggregations of walleye pollock. The remaining 12S ASVs annotated to two fish species, *Thaleichthys pacificus* and *Diaphus theta*; these species were at very low relative abundance (<1% of fish sequences) and we considered these to be incidental hits (i.e., non-target prey items) (Table 3).

**Table 3:** Summary of fish DNA sequences (12S rRNA gene) recovered from the feces of tagged humpback whale BCY0983.

Common name	Species	Prey DNA reads	% of reads
Walleye pollock	*Gadus chalcogrammus*	114,880	99.9
Eulachon	*Thaleichthys pacificus*	30	<1
California headlightfish	*Diaphus theta*	9	<1

The genetic analysis of marine invertebrate prey items produced 291,005 quality-filtered COI reads for diet analysis. However all but 124 of these sequences (99.9%) were annotated to the humpback whale. Despite low coverage these data inferred the presence of two marine invertebrate species in the fecal samples (*Euphausia pacifica* and *Thysanoessa raschii*), both of which are krill (order: Euphausiacea).

## Discussion

We describe the feeding behaviour of a solitary, subsurface-foraging humpback whale in Juan de Fuca Strait. We estimated feeding behaviour by three complementary methods: (*i*) whale-borne tag data, (*ii*) acoustic prey mapping, and (*iii*) fecal sub-sampling that was opportunistic, but provided insight into prey actually consumed by the whale within hours of data collection. An important limitation in this study was that we could not perform direct net sampling to confirm acoustic targets [18,19]; however, the distinctive structuring of the acoustic prey layers was similar to the backscatter attributed to walleye pollock recorded in the integrated surveys off the west coast of Vancouver Island and in the Strait of Georgia, including echo morphology, depth distribution, and single target attributes [47,48]. Pollock in British Columbia overlap with high densities of forage species (e.g., euphausiids) that are essential prey of fish including Pacific herring and Pacific salmon (*Oncorhynchus* spp.). These and other species are routinely sampled during Fisheries and Oceans Canada surveys to interpret the acoustic backscatter data, and to generate species-specific estimates of abundance or biomass. The characteristic layers in our study combined with high backscatter intensity at both frequencies, suggest that scattering can reasonably be assumed to be primarily from fish with gas-filled swim bladders and very likely walleye pollock. Although the size/age of pollock could not be determined without net sampling, the fecal sample from this whale revealed DNA sequences dominated by *Gadus* spp. and fish bones from juvenile pollock.

Our results show that the whale was likely feeding on walleye pollock, a semi-demersal schooling fish in the North Pacific that increasingly associates with the seafloor from juvenile to adult [66]. In Alaska, walleye pollock have extremely high biomass that sustains the largest commercial trawl fisheries off the west coast of North America (annual catch >1 million tonnes) [67]. In southern British Columbia, Canada, smaller populations of walleye pollock spawn off the west, north, and south coasts of Vancouver Island, including Queen Charlotte Sound and in the Strait of Georgia [66]. Juvenile pollock in British Columbia recruit to smaller-scale but commercially valued British Columbia trawl fisheries (coastwide total allowable catch ~4,000 tonnes in 2016), for which stock-assessment surveys have documented an increasing presence of humpback whales foraging around the survey transect lines. Our findings are the first confirmation of humpback whale pollock foraging in British Columbia that may increase where juvenile pollock aggregations occur in sufficient densities. From a management perspective, important prey species of humpback whales in British Columbia waters are not well understood [41]. The continued positive growth rate for the North Pacific humpback whale population (~8%/yr [38,68]) suggests that the overlap of foraging humpbacks, fishing vessels, and gear is likely to increase, along with the problems of gear entanglements, vessel strikes, and disruption of feeding during a narrow window of food acquisition that can reduce the likelihood of survival for humpback whales [41,44,69].

Humpback whale foraging is known to be opportunistic on zooplankton and schooling fish species up to 20–30 cm in length [14,32,70], including juvenile walleye pollock in Alaska [14,20]. Past investigations using high-resolution tags have found that lunge feeding rates correlate with the type, density, and depth of prey [10,12,16,23,71]. As predicted by optimal foraging theory, rorquals have been observed to increase the length of foraging dives with increasing prey depth to optimize the time spent foraging against the time lost recovering oxygen at the surface, and these longer foraging dives correspond to higher feeding rates [23]. However, for a constant prey depth, models predict that dive duration (and lunge rates) should increase with increasing prey density [16]. In line with these predictions, our solitary humpback whale had a higher lunge-feeding rate at the highest prey abundance and stopped feeding when prey density became limited. Near the end of the deployment, the whale also spent more time at the foraging depth during the lowest prey detections, but was not lunging anymore, presumably in search of dispersing prey. Accordingly, energy loss and oxygen consumption were likely minimized by the whale’s decision not to lunge when prey fell below a critical threshold [16,30,50]. The lowest prey detections also coincided with the whale’s shallowest ascending angles to the surface. Shallower ascents were possibly an additional foraging tactic to enhance the whale’s detection of prey that was dispersing in horizontal space [71].

The observed foraging behaviour in this study also exhibited kinematic diversity [32] during the course of the deployment. Generally, the relationship between peak lunge speed and pitch was consistent with previous studies that have described sequential, underwater lunge-feeding behaviours of rorquals in other regions (e.g., [32,50,65]), with the final fluke stroke in our study possibly signalling a strong head-lift that resembled fluking in the gyroscope signal (W. Gough, pers. comm.). Also, at the higher prey detections, a series of discreet and sequential lunges resembled the methodical profile of a krill-feeding rorqual [32], but then lunging became more variable as prey abundance also declined. Both the acoustic prey data and the fecal sample results pointed to walleye pollock as the dominant prey item, with little evidence of zooplankton. While the food digestion time of rorquals is possibly ~15 hr [72], and thus long enough for the whale to have consumed the pollock elsewhere, this animal is recognised to be particularly site-faithful to Juan de Fuca Strait and was documented in the area on the days prior to tagging. Echosounder and tagging technologies can only measure predator-prey interactions at the time of data collection, emphasizing the utility of including fecal sampling in rorqual feeding studies [63].

In terms of predator avoidance performance, pollock may be less evasive than schooling fish species like herring. For instance, a video analysis by Inoue et al. [73] found that when exposed to trawl fishing nets towed at ~4 knots of speed (2.0 m s^-1^) in water of ~10°C, some walleye pollock (20–30 cm in total length) had maximum burst swimming speeds of ≤3.5 m s^-1^ (with slower speeds at lower water temperatures), but that most pollock exhibited relatively unresponsive drifting behaviour to the approaching net. Laboratory data have also shown a decrease in swimming speeds of juvenile pollock (2–5 cm in total length) during prolonged exposure to live predatory juvenile sablefish (*Anoplopoma fimbria*) [74]. The authors hypothesized that juvenile pollock may fatigue or even acclimate to the persistent interaction with a predator [74]. Assuming, then, that our interpretation of the data is accurate, and although the locomotor cost of fish-feeding versus krill-feeding in rorquals is generally higher due to the greater evasive capacity of fish [31], shoaling pollock may require less energy per lunge and similar attack strategies as for krill feeding. The whale in our study was foraging alone, on likely juvenile pollock, by striking from below with stealthier approaches and higher engulfment speeds than trawling, and with a repetition at higher prey density that mimicked typical krill-feeding behaviour in the tag sensor data.

Widely ranging consumers in the ocean and on land must track short-term feeding opportunities across resource landscapes [75], and benefit from remembering the physical and biotic features of smaller components of their population habitat [76]. Rorquals are long lived and may patrol a network of previously profitable foraging ‘hotspots’ based on memory and personal experience [8], thus buffering against intraspecific competition and long-term climate variability [8,77]. Given the recovery of North Pacific humpback whales [69] and the strong site fidelity exhibited by individuals along the west coast of North America (e.g., [78]), individual specialisations in prey use are likely to arise [79] and can provide benefits in otherwise generalist populations [77,80]. Our observation of a humpback whale feeding alone on seasonally energy-dense fish like walleye pollock [81], which are abundant during Fisheries and Oceans Canada pelagic ecosystem surveys, suggests that this may be a profitable strategy. Additional short-term tagging of site-faithful feeding whales and concurrent acoustic prey sampling that includes multiple lower frequencies (e.g., 38 and 70 kHz for greater contrast for differentiating species’ reflectance), should reveal the generality of our findings, and provide further insights into individual-level differences in prey choice and habitat specialization. In the future, if pollock-foraging humpback whales were tagged concurrent with acoustic prey mapping, this would provide a valuable opportunity to determine if a pollock-foraging kinematic signature in bio-logging data is distinguishable. However, we would expect the tag sensor data to vary according to the density and composition of the pollock aggregations [32].

Vessel strikes involving foraging large whales are of increasing concern in British Columbia [36,41]. The risk of whale-vessel collisions is strongly influenced by the location, abundance, and type of prey encountered by individual whales [36,42]. In our study, the observed variability in relative prey biomass affected the feeding performance of the whale. Such fine-scale prey and feeding data from multiple different whales would allow a better understanding of the subtle effects foraging may have on the response behaviour of a whale to an approaching vessel, and assist with adaptive management as new foraging information becomes available.

## Supporting information

S1 Table Taxonomic assignment. (XLSX)

S1 Text DNA analysis methods. (DOCX)
