# Correction: Integrating technologies provides insight into the subsurface foraging behaviour of a humpback whale (*Megaptera novaeangliae*) feeding on walleye pollock (*Gadus chalcogrammus*) in Juan de Fuca Strait, Canada

**DOI:** 10.1371/journal.pone.0306841

**Published:** 2024-07-03

**Authors:** The PLOS ONE Staff

## Notice of Republication

This article was republished on June 24, 2024, to correct errors in Tables 1 and 3. The publisher apologizes for the errors. Please download this article again to view the correct version. The originally published, uncorrected article and the republished, corrected articles are provided here for reference.

## Supporting information

S1 File Originally published, uncorrected article. (PDF)

S2 File Republished, corrected article. (PDF)
