# Supplementary material for: Integrating technologies provides insight into the subsurface foraging behaviour of a humpback whale (Megaptera novaeangliae) feeding on walleye pollock (Gadus chalcogrammus) in Juan de Fuca Strait, Canada
Source: PLoS One. 2023 Mar 6;18(3):e0282651. doi: 10.1371/journal.pone.0282651 (PMC9987809; doi:10.1371/journal.pone.0282651)
Supplement: S1 Text — (DOCX) [file pone.0282651.s002.docx]

# S1 Text. DNA analysis methods

The fecal sample from humpback whale BCY0983 was processed with 16 other humpback fecal samples as part of a larger, collaborative humpback fecal study for whales feeding in southern BC. All fecal samples were initially vortexed for 10s. We then subsampled 1,000 µl of stool mixed with ethanol, which was transferred to a clean Eppendorf tube (1.5 mL) for DNA extraction. The tubes centrifuged for 1 min at 4,500 x *g*, and the ethanol was removed by pipetting without disturbing the stool in the bottom of the tube. Immediately after removing the ethanol, 1 mL of InhibitEX buffer (Qiagen) was added to the stool sample. Genomic DNA was then extracted from all fecal samples using the QIAamp Fast DNA Stool Mini Kit (Qiagen) following the manufacturer’s protocols. A DNA extraction blank (negative control) was processed at the same time with the biological samples to test for cross contamination.

We used a metabarcoding approach to estimate the diversity of fish and marine invertebrates in each fecal sample. Fish were targeted using a short section of the MiFish region of the 12S rRNA gene (MiFish-U; Miya et al., 2015). Invertebrates were targeted using a short section of the Cytochrome c Oxidase subunit I gene (COI) with the primers *mlCOIintF* and *dgHCO2198* (2,3). A two-step polymerase chain reaction (PCR) (4) was used to amplify the target regions (first round PCR), and to attach Illumina adapters and unique barcodes to the amplicons for sequencing (second round PCR). A negative control sample was included in all PCR reactions for both genes.

The first round PCR was carried out with 3 replicate PCRs per sample, each with a 25 µl reaction volume containing 5.5 µl sterile Nuclease-Free water, 0.6 µl of each primer (10 µM), 2 µl BSA (10 mg/mL) (NEB), 12.5 µl 2X Taq (Froggabio), and 2 µl template, and with the following thermal cycling conditions: polymerase chain activation of 95˚C for 5 min, followed by 40 cycles of denaturation at 95˚C for 30 sec, annealing at 63˚C (12S rRNA) or 52˚C (COI) for 30 sec, and extension of 72˚C for 45 sec, with a final extension of 72˚C for 5 min. The PCR replicates were then pooled, and excess primers and unspecific fragments of <300 bp were removed with a SPRI cleanup, following the manufacturer’s protocols. Samples were then resuspended with 25 µl of sterile nuclease free (NF) water for the second round PCR.

The second PCR reaction volume also contained 2.5 µl of each primer, 12.5 µl of 2X Taq (Froggabio) and 2.5 µl template (first PCR product), with different combinations of indices (i5 and i7) used to assign a unique identification to each sample. This PCR used the following thermal conditions: polymerase activation of 95˚C for 3 min, 8 cycles of denaturation at 95˚C for 30 sec, annealing at 55˚C for 30 sec, and extension of 72˚C for 30 sec, with a final extension of 72˚C for 5 min, followed by a second SPRI beads cleanup, and resuspension with 25 µl of NF water. DNA contents were then quantified using a Qubit dsDNA High-Sensitivity assay kit. Samples were equimolar pooled using 40 ng/sample, and the libraries sequenced using the Illumina MiSeq V2 chemistry (500 cycles) at the Hakai Institute genomics facility with a separate library for each gene.

Paired-end, fastq-formatted reads were processed using the R package DADA2 (5), and primers were trimmed using the command-line program cutadapt v2.10 (6). After learning error rates, dereplication, sample inference, and read merging with the default parameters in DADA2, we removed samples that had fewer than 50 true amplicon sequence variants (ASVs) present. Also, if ASVs were present in only a single sample with lower than 0.001 relative abundance, then that sample was removed (hereafter referred to as ‘singleton ASVs’). Bimeras (chimera sequences with two identifiable sources) were removed using the default parameters in DADA2.

Taxonomy assignments for both the COI and 12S libraries were derived using the National Center for Biotechnology (NCBI) BLASTN 2.10.0+ program. BLAST output was limited in this analysis to hits that had ≥96% similarity to the query, ≥50% query coverage, and an e-value of ≥10^-5^. The lowest common ancestor (LCA) taxonomy strings for each ASV was obtained using the Galaxy Tool LCA pipeline (https://github.com/naturalis/galaxy-tool-lca). For each ASV, the top scoring blast hit was applied without using LCA if it had ≥98% similarity with the query, otherwise LCA was employed to determine a consensus taxonomy.

The 12S data were then filtered to include only ASVs that were assigned to either the humpback whale or the two major classes of fishes: Actinopteri (ray-finned) and Chondrichthyes (cartilaginous). We also removed 12S ASVs that were mis-annotated to species that live outside of the geographical range of this study. We then collapsed the data when multiple ASVs annotated to the same species. For the COI data, we removed ASVs that lacked a phylum-level assignment and then retained only the humpback whale and marine invertebrate taxa (vertebrates and terrestrial arthropods were removed). Any reads detected in the negative controls were subtracted from the fecal sample data. For both genes we collapsed the data when multiple ASVs annotated to the same species. Humpback whale sequences were retained from both datasets in order to assess the relative coverage of the whale compared to prey taxa.

**REFERNCES**

1. Miya M, Sato Y, Fukunaga T, Sado T, Poulsen JY, Sato K, et al. MiFish, a set of universal PCR primers for metabarcoding environmental DNA from fishes: Detection of more than 230 subtropical marine species. R Soc Open Sci. 2015; 2. doi:10.1098/rsos.150088

2. Meyer CP. Molecular systematics of cowries (Gastropoda: Cypraeidae) and diversification patterns in the tropics. Biol J Linn Soc [Internet]. 2003;79:401–459. <https://doi.org/10.1046/j.1095-8312.2003.00197.x>

3. Leray M, Yang JY, Meyer CP, Mills SC, Agudelo N, Ranwez V, et al. A new versatile primer set targeting a short fragment of the mitochondrial COI region for metabarcoding metazoan diversity: Application for characterizing coral reef fish gut contents. Front Zoo. 2013;10. doi:10.1186/1742-9994-10-34

4. Bourlat SJ, Haenel Q, Finnman J, Leray M. Preparation of amplicon libraries for metabarcoding of marine eukaryotes using Illumina MiSeq: The dual-PCR method. In: Bourlat SJ, editor. Mar Genomics: Methods Protoc. 2016;1452:197–207. https://doi.org/10.1007/978-1-4939-3774-5_13

5. Callahan BJ, McMurdie PJ, Rosen MJ, Han AW, Johnson AJA, Holmes SP. DADA2: High-resolution sample inference from Illumina amplicon data. Nat Methods. 2016;13:581–583. doi:10.1038/NMETH.3869

6. Martin M. Cutadapt removes adapter sequences from high-throughput sequencing reads. EMBnet J [Internet]. 2011;17:10-12. <https://doi.org/10.14806/ej.17.1.200>
